# Translocation of Non-Canonical Polypeptides into Cells Using Protective Antigen

**DOI:** 10.1038/srep11944

**Published:** 2015-07-16

**Authors:** Amy E. Rabideau, Xiaoli Liao, Gizem Akçay, Bradley L. Pentelute

**Affiliations:** 1 Department of Chemistry, Massachusetts Institute of Technology, 77 Massachusetts Avenue 18-596, Cambridge, MA 02139

## Abstract

A variety of pathogenic bacteria infect host eukaryotic cells using protein toxins, which enter the cytosol and exert their cytotoxic effects. Anthrax lethal toxin, for example, utilizes the membrane-spanning translocase, protective antigen (PA) pore, to deliver the protein toxin lethal factor (LF) from the endosome into the cytosol of cells. Previous work has investigated the delivery of natural peptides and enzymatic domains appended to the C-terminus of the PA-binding domain of lethal factor (LF_N_) into the cytosol via PA pore. Here, we move beyond natural amino acids and systematically investigate the translocation of polypeptide cargo containing non-canonical amino acids and functionalities through PA pore. Our results indicate translocation is not perturbed with alterations to the peptide backbone or side-chain. Moreover, despite their structural complexity, we found that the small molecule drugs, doxorubicin and monomethyl auristatin F (MMAF) translocated efficiently through PA pore. However, we found cyclic peptides and the small molecule drug docetaxel abrogated translocation due to their large size and structural rigidity. For cargos that reached the cytosol, we demonstrated that each remained intact after translocation. These studies show PA is capable of translocating non-canonical cargo provided it is in a conformational state conducive for passage through the narrow pore.

Nature has evolved several types of translocation systems for delivery of toxic proteins into host cells^1^. Certain pathogenic bacteria such as *Vibrio cholerae*, *Corynebacterium diphtheria,* and *Bacillis anthracis* infect host cells with cholera, diphtheria, and anthrax toxin, respectively^2^. These protein toxins access the cytosol by two main pathways: transport through a translocase from the host cell endosome or retrograde endocytosis from the endosome to the Golgi and endoplasmic reticulum^2^. Anthrax toxin is one example that utilizes a translocase to deliver protein toxins from the endosome into the cell cytosol^3^.

Anthrax lethal toxin is comprised of two proteins, protective antigen (PA) and lethal factor (LF)^3^. The pore-forming protein, PA_83_ binds to an anthrax toxin receptor on the host cell surface and is then proteolytically activated by furin protease to give PA_63_^4^, triggering oligomerization of PA_63_ into a heptameric^5^ or octameric^6^ pre-pore. LF binds to the pre-pore through the 263-residue N-terminal domain (LF_N_) with nanomolar affinity^7^. Up to three^8^ or four^6^ molecules of LF can bind to the heptameric or octameric pre-pore, respectively. The entire complex is then endocytosed and the low pH triggers a conformational rearrangement of the pre-pore to form a pore that spans through the endosomal membrane^9^, initiating translocation of LF into the cytosol^10^,^11^.

Biophysical and biochemical experiments of LF translocation through the cation-selective PA pore have elucidated the translocation mechanism into the cell cytosol^12^,^13^. The working model points toward a number of key factors that govern translocation through PA pore and subsequent escape from the endosome:

- PA pre-pore undergoes conformational rearrangement to form a pore in the endosomal membrane^5^,^14^.
- Charge state-dependent Brownian ratchet drives translocation through the PA pore^12^.
- Protein cargo partially unfolds in the acidic endosome prior to translocation^15^,^16^.
- Cargo passes through the PA conduit of approximately 12 Å diameter^13^,^17^.

Taking advantage of these drivers of translocation, researchers have investigated the delivery of various protein cargos fused to the C-terminus to LF_N_ through PA pore. Enzymes or enzymatic domains such as β-lactamase^18^,^19^, *Pseudomonas* exotoxin A, *Diphtheria* toxin A chain^20^,^21^, actin cross-linking domain from the *Vibrio cholerae* RTX toxin^22^, *Shiga* toxin^23^, and *Legionella pneumophila* flagellin protein^24^ as well as antibody mimic proteins^25^ have been delivered into cells using the PA/LF_N_ system. These studies showed that the PA pore is efficient in transporting naturally occurring or recombinantly expressed proteins. However, the question of whether PA pore can accommodate and transport molecules beyond natural polypeptides has yet to be investigated. One example has shown the effects on translocation when D-amino acids and cysteic acid are incorporated at the N-terminus of LF_N_^26^. Recently, Rabideau, *et al*. demonstrated that mirror image polypeptides and proteins^27^ on the C-terminus of LFN can translocate efficiently through PA pore. Some non-natural molecules, including small molecule drugs and polypeptides with backbone or side chain modifications, have attracted research interest because of their potential as therapeutics. Investigating the translocation of these non-canonical polypeptides would not only aid in their cytosolic delivery, but also provide insight into the mechanism and promiscuity of the PA translocase.

In the present study, we investigated the translocation of various non-canonical polypeptide moieties through the PA translocase (Fig. 1a). We designed our experiments to seek answers to three questions: 1, Do non-natural amino acids affect translocation through PA pore? 2, Can PA pore still tolerate cargos containing non-natural peptide backbones? 3, Can a constrained polypeptide or rigid natural product pass through the 12 Å pore? To carry out these investigations, we leveraged the use of sortase A (SrtA)-mediated ligation. The non-canonical polypeptide cargos were synthesized with an N-terminal oligoglycine motif and attached to the C-terminus of LF_N_-DTA using the LPXTG tag and SrtA^28^ to give LDn constructs (Fig. 1b). DTA (A chain of diphtheria toxin) serves as a reporter of translocation since it inhibits protein synthesis upon entry into the cytosol^5^,^23^,^29^. From our investigations, we found peptides containing minor modifications to the backbone or amino acid side chains, peptidomimetic monomethyl auristatin F (MMAF), and the small molecule drug, doxorubicin translocate efficiently. In contrast, cyclic peptides and the small molecule drug, docetaxel caused disruption to the translocation process. Our findings support the hypothesis that the chemical composition of the cargo does not inhibit the Brownian ratchet provided the cargo is able to adopt a conformational state in which it can pass through the narrow pore.

## Results

### Translocation of non-canonical polypeptide cargos with backbone or side chain modifications

**Figure 2:**
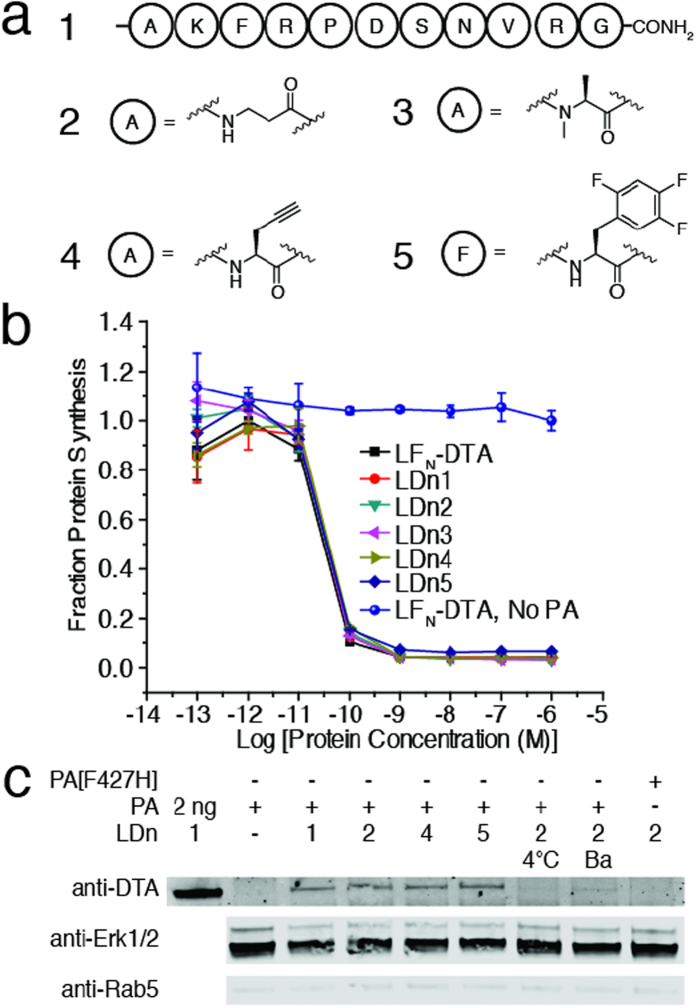
Translocation of non-canonical peptides **a**) Peptides 1–5 containing non-canonical side-chain modifications were ligated to LF_N_-DTA using SrtA to form LDn1–5 **b**) Protein synthesis inhibition assay in CHO-K1. Ten-fold serial dilutions of LDn1–5 and LF_N_-DTA were added to CHO-K1 in the presence of 20 nM PA and incubated for 30 minutes at 37 °C and 5% CO_2_ then washed with PBS. Treated cells were chased with ^3^H-Leu in Leu-free medium for 1 hour then washed and read using a scintillation counter. **c**) CHO-K1 cells were treated with 100 nM LDn’s in the presence of 20 nM PA for 12 hours. The cells were lifted and the cell surface was trypsin digested for 5 minutes then subsequently washed with PBS. The cell cytosol was extracted using 50 μg mL^−1^ digitonin for 10 min on ice. The cytosol extraction was analyzed by western blot. The following controls were also analyzed: incubation at 4 °C (instead of 37 °C), incubation with Bafilomycin A1, and use of mutant PA, PA[F427H]. Cropped blots are used in western blot data (c).

We first investigated the translocation of peptides containing either backbone or side chain modifications. We chose β-alanine and N-methyl-alanine for backbone modifications, and propargylglycine and 2,4,5-tri fluoro-phenylalanine for side chain modifications. These modifications were incorporated into a model peptide containing all natural amino acids, G_5_-AKFRPDSNVRG (Supplementary Table 1). These peptides were sortagged onto LF_N_-DTA to give LDn1–5 (Fig. 2a; Supplementary Table 2). We first used a protein synthesis inhibition assay to analyze translocation efficiency of each construct^5^,^23^,^29^. For this assay, ten-fold serial dilutions of each purified conjugate were added to CHO-K1 cells in the presence of 20 nM PA and incubated for 30 minutes. Cells were washed three times with PBS then incubated with medium supplemented with ^3^H-Leu for 1 h. After incubation, medium was removed, cells were washed, and ^3^H-Leu incorporation was measured by a scintillation counter to determine the translocation efficiency of each cargo. According to Fig. 2b, each non-canonical cargo (LDn2–5) translocated as efficiently as the controls (Supplementary Table 3), LF_N_-DTA and LDn1, indicating that these peptides, which contain backbone and side chain modifications can be appended to the C-terminus of LF_N_-DTA without major interference to the translocation process.

We further analyzed the cytosolic presence of the non-canonical peptide conjugates using digitonin extraction and western blot. CHO-K1 cells were treated with 100 nM LDn1–5 in the presence of 20 nM PA for 12 hours to allow for multiple rounds of endocytosis and translocation. After trypsin digestion of surface bound materials and washing with PBS, the cytosolic proteins were extracted using a lysis buffer containing 50 μg mL^−1^ digitonin, a mild detergent used to permeabilize only the plasma membrane^30^. The proteins extracted by digitonin were analyzed by western blot immunostained with anti-Erk1/2 (a cytosolic marker) and anti-Rab5 (an early endosome marker) to confirm the presence of only cytosolic proteins. Using immunostaining by anti-DTA antibody, we observed bands corresponding to LDn2, 4, and 5 with intensities similar to that of LDn1 (Fig. 2c). We also analyzed the total cell lysate using a buffer containing 1% NP-40 and observed comparable band intensities detected by the anti-DTA antibody (Supplementary Figure 1). This data further confirmed the results from the protein synthesis inhibition assay, indicating that these non-natural modifications did not interfere with the translocation through PA pore. In addition, we performed control experiments with LDn2 by incubating the cells at 4 °C instead of 37 °C, incubating with an inhibitor of endosomal acidification (Bafilomycin A1), or using a mutant PA (PA[F427H]) that binds LF_N_ but arrests translocation. In all three cases, we observed no band corresponding to LDn2, indicating the translocation process followed the same mechanism as LF_N_-DTA, which requires active endocytosis, an acidic endosome, and a functional PA (Fig. 2c).

### Translocation of cyclic peptides

**Figure 3:**
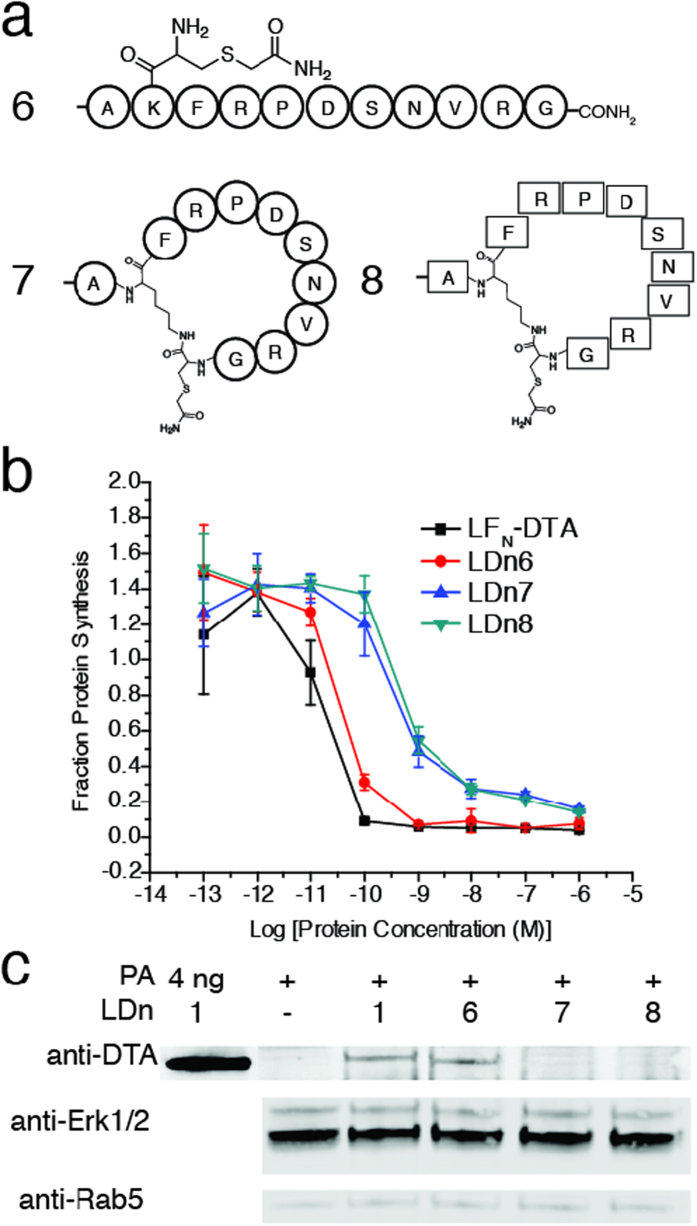
Translocation of cyclic peptides **a**) Linear control peptide and L and D cyclic peptides to form LDn6–8, respectively **b**) CHO-K1 were treated with ten-fold serial dilutions of LDn6–8 and LF_N_-DTA in the presence of 20 nM PA for 30 minutes. The same experimental conditions were used as previously described. **c**) CHO-K1 were treated with 100 nM LD6–8 in the presence of 20 nM PA for 12 hours then subjected to cytosolic extraction for western blot, as previously described. Cropped blots are used in western blot data (c).

We expanded our library of non-canonical peptides under investigation to include cyclic peptides. In comparison to linear peptides, cyclic peptides have increased structural rigidity and proteolytic stability, making them attractive therapeutic candidates^31^. We cyclized L and D forms of the model peptide (1) using native chemical ligation (Fig. 3a). As demonstrated in Supplementary Scheme 1, we installed a Cys residue on the ε-amino group of the Lys residue of the peptide thioester in both L (7) and D (8) forms. The L model peptide containing an alkylated Cys residue at the Lys position served as a linear control (6) for the translocation of the cyclic peptides. Each peptide was sortagged onto LF_N_-DTA then analyzed using both the protein synthesis inhibition and western blot assays. We found that there was a substantial reduction in translocation efficiency for both LDn7 and LDn8, as compared to LF_N_-DTA and LDn6 (Fig. 3b; Supplementary Table 3). Furthermore, we treated CHO-K1 with 100 nM LDn6–8 in the presence of 20 nM PA overnight then analyzed cytosolic delivery in digitonin extracted materials. The western blot immunostained with anti-DTA showed no detectable band for LDn7 and LDn8, while the linear control peptides LDn1 and LDn6 showed similar band intensity (Fig. 3c). A small amount of LDn7 and LDn8 was detected in the total cell lysate (Supplementary Figure 2), indicating some cargos were trapped in endosomes. The western blot result was consistent with the protein synthesis assay results, showing that translocation was substantially reduced for the cyclic peptide conjugates, LDn7 and LDn8, compared to the linear peptide controls.

### Translocation of complex small molecules

**Figure 4:**
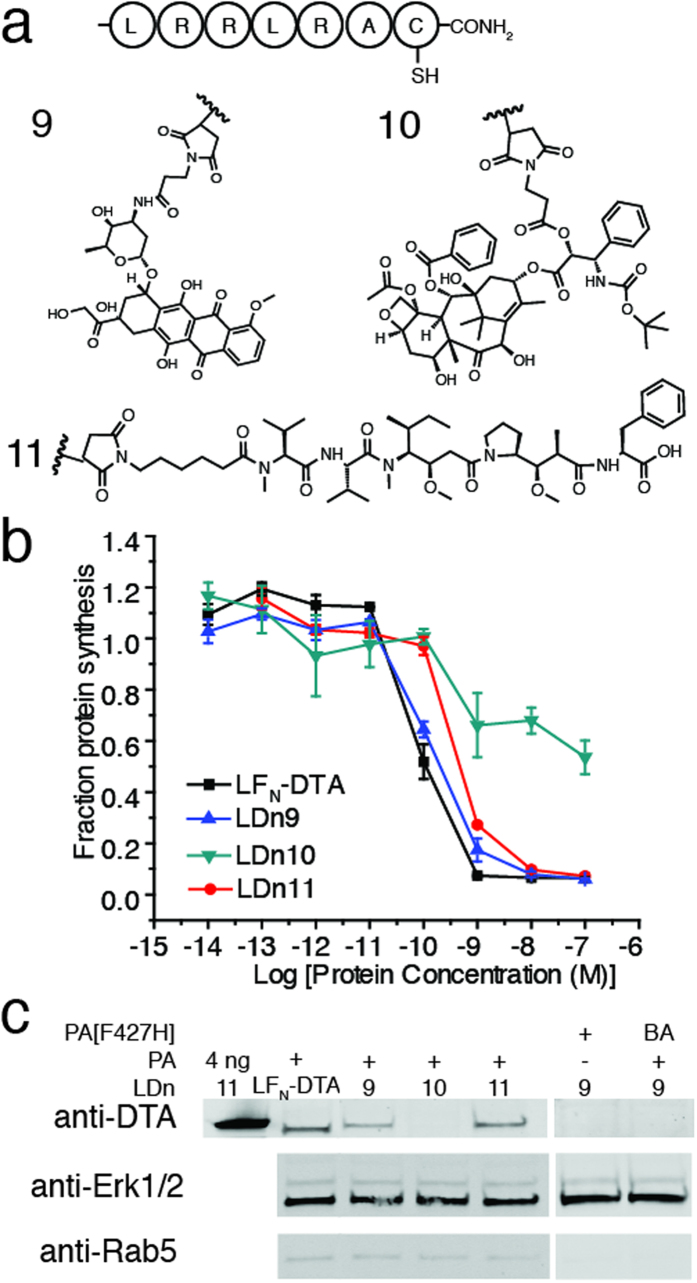
Translocation of small molecules **a**) Small molecules doxorubicin, docetaxel, and monomethyl auristatin F (MMAF) were conjugated at the cysteine residue of G_5_-LRRLRAC then ligated to LF_N_-DTA to form LDn9–11. **b**) CHO-K1 cells were treated with ten-fold serial dilutions of LDn9–11 and LF_N_-DTA in the presence of 20 nM PA for 30 minutes. The same experimental conditions were used as previously described. **c**) CHO-K1 cells were treated with 100 nM LDn9–11 in the presence of 20 nM PA for 12 hours then digitonin extracted and run on western blot, as previously described. Cropped blots are used in western blot data (c).

We next investigated the ability of PA to translocate complex, cytotoxic small molecules that are frequently used in antibody-drug conjugates: doxorubicin, docetaxel, and monomethyl auristatin F (MMAF)^32^. Direct translocation of these small molecules into the cytosol would be of great interest for the delivery of impermeable chemotherapeutics or other small molecule drugs. These small molecules were conjugated through the Cys residue of the peptide, G_5_-LRRLRAC, using a maleimide functional group (Fig. 4a; Supplementary Scheme 2). The peptide-small molecule products were then sortagged onto LF_N_-DTA (LDn9–11, respectively) for investigation of translocation through PA pore. Again, we used the protein synthesis inhibition assay and found that LDn9 and LDn11 translocated as efficiently as the LF_N_-DTA control, while translocation was completely arrested for LDn10 (Fig. 4b; Supplementary Table 3). We then treated the cells with 100 nM LDn9–11 in the presence of 20 nM PA overnight and analyzed cytosolic delivery by immunostaining with anti-DTA antibody. We observed similar band intensities for LDn9 and LDn11 as compared to LF_N_-DTA, while no band was detectable for LDn10 (Fig. 4c). We also analyzed the total cell lysate and observed comparable band intensities with the anti-DTA antibody (Supplementary Figure 2). The western blot results were consistent with protein synthesis inhibition results, which suggest that the PA pore was efficient in accommodating cytotoxic molecules whose structures were either similar to (i.e. MMAF) or distinct from (i.e. doxorubicin) polypeptides. We hypothesized that the three-dimensional size of the molecule was a major factor that contributed to translocation efficiency. Therefore, we estimated the longest linear distance for doxorubicin and docetaxel based on published crystal structures. Despite different possible conformational arrangements, we found that the longest linear distance for doxorubicin in the crystal structure to be about 10.3 Å^33^, while that of docetaxel was about 13.5 Å^34^, which is close to the estimated PA pore size (12 Å)^17^. Taken together, these data suggested that PA pore was promiscuous enough to translocate cytotoxic small molecules, but with a size limitation due to the inherent size of the pore.

### Translocation of intact cargo

**Figure 5:**
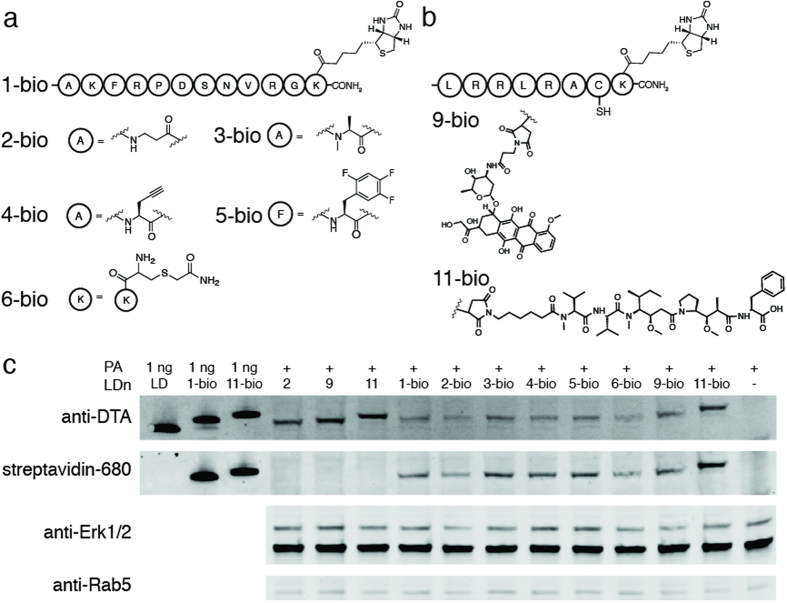
Translocation of C-terminally biotinylated cargo **a**) Non-canonical peptides 1–6 were modified to contain biotin on a C-terminal lysine residue (1-bio to 6-bio) then ligated to LF_N_-DTA to form LDn1-bio to 6-bio **b**) Peptides containing the small molecules, doxorubicin and monomethyl auristatin F (MMAF) were modified to contain biotin on a C-terminal lysine residue (9-bio and 11-bio, respectively) then ligated to LF_N_-DTA to form LDn9-bio and LDn11-bio **c**) CHO-K1 cells were treated with 100 nM LDn2, LDn9, LDn11, LDn1-bio to LDn6-bio, LDn9-bio, and LDn11-bio in the presence of 20 nM PA for 12 hours then digitonin extracted and run on western blot, as previously described. Cropped blots are used in western blot data (c).

Our studies indicate a variety of non-canonical polypeptide cargo can translocate through PA pore into the cell cytosol. To verify that the protein conjugates containing non-canonical polypeptide cargos passed through the pore and into the cytosol without any truncations, we analyzed the translocation of intact cargos. For this analysis, we installed a biotin at the C-terminus of each non-canonical polypeptide cargo that translocated efficiently (Fig. 5a,b). The C-terminal biotin provided a small tag for western blot detection of intact cargos, which were sortagged onto LF_N_-DTA as previously described (LDn1-bio to LDn6-bio, LDn9-bio, and LDn11-bio). Translocation of the biotinylated conjugates was achieved by treating CHO-K1 cells with 100 nM LDn1-bio to LDn6-bio, LDn9-bio, and LDn11-bio in the presence of 20 nM PA for 12 hours. The cells were subjected to the same harvest and digitonin lysis conditions as previously described. Prior to western blot analysis, the SDS-PAGE gel was run for approximately twice as long as the previous experiments in order to differentiate the shifts in molecular weights of the conjugates containing intact cargo. The western blot in Fig. 5c was stained with anti-DTA (subsequently stained with goat anti-mouse IRdye 800CW secondary antibody) and streptavidin IRdye 680LT. The co-staining of anti-DTA and streptavidin for all the biotinylated conjugates (LDn1-bio to LDn6-bio, LDn9-bio, and LDn11-bio) confirmed the presence of the intact protein conjugates in the cytosol of cells. Since doxorubicin and MMAF were conjugated through non-amide linkages (i.e. maleimide) we relied on shifts in molecular weight in the western blot to confirm that the conjugates remained intact after translocation. As demonstrated in Fig. 5c, the shifts in molecular weight for the biotinylated conjugates corresponded with the 1 ng loading controls (LF_N_-DTA, LDn1-bio, LDn11-bio) as well as the non-biotinylated translocated materials (LDn2, LDn9, and LDn11) indicating that the translocated material was indeed intact.

## Discussion

In this paper, we investigated whether PA pore could efficiently transport non-canonical polypeptides into the cell cytosol. We used two separate assays to evaluate translocation efficiency. The protein synthesis inhibition assay based on DTA activity is widely used in the anthrax toxin field to probe translocation, enabling direct comparison to the previous reports. We also used cytosolic extraction by digitonin and western blot analysis to further investigate delivery of the cargos in the cytosol.

Based on these two assays, we found that several variants did not perturb translocation through PA pore. These peptides contain backbone modifications, or side chain modifications that can be used to increase proteolytic stability (e.g. β-alanine, N-methyl-alanine), binding affinity (e.g. fluoro-phenylalanine), or serve as chemical handles (e.g. alkyne or biotin groups) for follow-up analysis. We also demonstrated the translocation of small molecule drugs such as doxorubicin and MMAF through PA pore. Altogether these examples indicate that PA pore is relatively promiscuous in terms of the substrates that can be translocated. Once unfolding and translocation is initiated by the interaction of LF_N_ with PA pore in the acidic endosome, the pore is able to accommodate the trailing segment, with high tolerance for chemical modifications.

The digitonin extraction and western blot analysis not only confirmed the cytosolic delivery of these materials through PA pore, but also supported the reported translocation mechanism. The requirement of endocytosis, endosome acidification, and active PA pore was indicated by the control experiments where no bands were detected using 4 °C, Bafilomycin A1, or PA [F427H].

Model cyclic peptides and complex small molecules such as docetaxel perturbed translocation through PA pore, as indicated by the substantial reduction or abolishment of protein synthesis inhibition. We hypothesize that the size of the molecules were responsible for arresting translocation. For the cyclic peptides, although the structures are unknown, the potential rigidity and large size could perturb translocation. Furthermore, inhibited translocation is independent of stereochemistry, as evidenced by the translocation of the D cyclic peptide cargo. Although there was some detectable protein synthesis inhibition activity with the cyclic peptides, we observed no material by western blot of the cytosolic fractions. We note that the protein synthesis inhibition assay is a more sensitive assay since its readout is correlated with the activity of the DTA enzyme, which turns over at very low concentrations, while the western blot approach does not allow us to detect very low levels of protein. Regardless of the detection methods, both demonstrated that these large and constrained cargos had difficulty passing through PA pore. These results provide additional evidence to support the translocation mechanism, where cargos need to be in an extended confirmation to pass through the pore; large and rigid molecules cannot efficiently pass through the pore. This result also provides design principles for different cargos of interest.

Moreover, we analyzed the translocation of intact polypeptide cargo. Non-canonical polypeptide cargo, which were found to translocate efficiently were biotinylated on the C-terminus. After translocation and digitonin extraction, the western blot was analyzed for the presence of DTA as well as biotin using streptavidin labeled with an IRdye. Co-staining of DTA and streptavidin for each conjugate as well as band shifts corresponding to the correct molecular weights indicated that the protein conjugates containing non-canonical polypeptide cargos remained intact after translocation through PA pore into the cytosol.

Nature evolved to utilize 20 amino acids to make up proteins. Recently, there has been great interest to incorporate non-canonical amino acids into polypeptides in order to enhance biological properties or to study biological processes. For our study, we asked the basic question of whether these non-natural amino acids could be efficiently translocated into the cell cytosol through the anthrax toxin PA pore. Indeed, we found the PA translocase still functions despite the presence of non-canonical cargo.

## Methods

### Peptide synthesis

Select peptides were synthesized under fast-flow coupling/deprotection conditions using Fmoc chemistry protocols for SPPS^35^. All the peptides were synthesized on aminomethyl resin with the rink amide linker at a 0.1 mmol scale. After synthesis, peptides were cleaved and side chain protecting groups were removed by TFA cleavage. A typical TFA cleavage solution contained TFA/H_2_O/EDT/TIPS (94/2.5/2.5/1 *v/v*). Resin was treated with TFA cleavage solution for 2 hours at room temperature, followed by N_2_ bubbling until dryness. The crude peptide was recovered by precipitation with cold ethyl ether as a white powder, then dissolved in 50% aqueous acetonitrile containing 0.1% TFA and lyophilized.

### LCMS analysis

The purity of all peptides and proteins were analyzed by high resolution LC-MS (Agilent 6520 Accurate-Mass quadrupole time-of-flight liquid chromatography mass spectrometry system). The samples were analyzed on an Agilent Zorbax 300SB C_3_ column (2.1 × 150 mm, 5 μm) using 0.8 mL min^−1^ and a linear gradient of 1–61% or 5–65% B’ over 9 min (A’: water + 0.1% formic acid; B’: acetonitrile + 0.1% formic acid). The observed mass was reported by averaging the major peak in the total ion current (TIC). For each protein, the charge state series was deconvoluted (maximum entropy setting) using Agilent MassHunter Bioconfirm.

### Preparative, semi-preparative, and analytical RP-HPLC

Crude peptides were purified by reverse phase-HPLC. The peptides were dissolved in 99:1 or 95:5 A:B (A: water + 0.1% TFA; B: acetonitrile + 0.1% TFA) and 6 M guanidine hydrochloride was added depending on the solubility of the peptide. For preparative RP-HPLC purification, we used an Agilent Zorbax SB C_18_ column (21.2 × 250 mm, 7 μm) at a flow rate of 10 mL min^−1^ at 1–41% or 5–45% B over 80 min. For semi-preparative RP-HPLC purification, we used an Agilent Zorbax SB C_18_ column (9.4 × 250 mm, 5 μm) at a flow rate of 5 mL/min over the same gradient. UV absorbance was monitored at 214 nm. Purity of the fractions was analyzed by MALDI or LC-MS. HPLC fractions from preparative or semi-preparative HPLC were spotted with MALDI matrix alpha-cyano-4-hydroxycinnamic acid (CHCA) in 50% A: 50% B and checked for the correct molecular masses. The analytical RP-HPLC Agilent C_18_ Zorbax SB column (2.1 × 150 mm, 5 μm) was used to confirm the purity of fractions at a flow rate of 0.5 mL/min over a linear gradient of 1–51% B over 12 min. Analytical HPLC UV absorbance traces were measured at 214 nm.

### Conjugation of docetaxel, doxorubicin, and MMAF to G_5_-LRRLRAC

Doxorubicin-maleimide (16 mg) or docetaxel-maleimide (30 mg) was added to a 16.5 mM solution of peptide G_5_-LRRLRAC in DMF to a final concentration of 16.5 mM (1:1). The reaction mixtures were allowed to stir at 36 °C for 5 hours, followed by additional 10 hours at room temperature, at which time LCMS analysis indicated consumption of the starting peptide and formation of G_5_-LRRLRAC(doxorubicin) and G_5_-LRRLRAC(docetaxel) peptide conjugates. The products were purified by semi-preparative RP-HPLC to give 35 mg (82% yield) of G_5_-LRRLRAC(doxorubicin) and 50 mg (75% yield) of G_5_-LRRLRAC(docetaxel).

MMAF was also ligated to G_5_-LRRLRAC using 2 mg maleimide-MMAF (20 mM) in DMSO and 20 mM G_5_-LRRLRAC (2.5 mg). Reaction was incubated at room temperature for 2 h. LCMS indicated consumption of the peptide starting material and formation of G_5_-LRRLRAC(MMAF) product. The product was purified by semi-preparative RP-HPLC to give 4.8 mg (86% yield).

Similar coupling strategies were utilized for the synthesis of G_5_-LRRLRAC(doxorubicin)K(biotin) and G_5_-LRRLRAC(MMAF)K(biotin) in which G_5_-LRRLRACK(biotin) was used as a starting material.

### Cyclization of linear peptide using native chemical ligation

Cyclization of the L-linear peptide precursor peptide was performed by conversion of the thiazolidine residue into a Cys residue and subsequent native chemical ligation (NCL), according to Supplementary Scheme 1. To convert the thiazolidine residue into a Cys residue, 30 mg peptide (2 mM) was dissolved in 0.2 M sodium phosphate buffer containing 6 M guanidine·HCl, 20 mM TCEP·HCl, and 0.2 M methoxyamine hydrochloride (MeONH_2_·HCl). The reaction was incubated at pH 4.0 at RT for 6 h. In the same pot^36^, NCL was performed to cyclize the peptide by adding 10 mM 4-mercaptophenylacetic acid (MPAA) and incubating at pH 7.0 for 1 h at RT^37^. The cyclized product was purified by semi-preparative RP-HPLC. Pure fractions that were confirmed by MALDI and analytical RP-HPLC were combined and lyophilized to give 7 mg, 4.3 μmol (26.1% yield). The Cys residue in the cyclized peptide was alkylated using 50 mM bromoacetamide and 20 mM TCEP·HCl in 0.2 M sodium phosphate buffer at pH 7.0 for 15 min at RT. Once complete, the reactions were quenched with 100 mM 2-mercaptoethanesulfonate (MESNa) and purified by semi-preparative RP-HPLC. Fractions containing the pure, alkylated L-cyclic peptide were combined and lyophilized to give 2.3 mg, 1.4 μmol (31.7% yield). Cyclization of the D peptide was performed using the same method as described for the L cyclic peptide. The pure D cyclic peptide yield was 6.0 mg, 3.7 μmol (19.1% yield) and the alkylated D cyclic peptide yield was 2.2 mg, 1.3 μmol (35.4% yield).

### Sortase-mediated ligation

Sortase A was used to ligate peptides containing the N-terminal oligoglycine motif to LF_N_-DTA-LPSTGG (LDn) as previously described. *Staphylococcus aureus* SrtA (P94S/D160N/K196T; SrtA*) evolved by Chen, *et al.* was used for our sortase-mediated ligations^38^. The purity of the ligated product was analyzed by LCMS. Concentrations of the ligated products containing non-canonical functionalities were determined using Bradford assay.

### Protein synthesis inhibition assay

CHO-K1 cells were maintained in F-12K medium containing 10% (v/v) fetal bovine serum, 100 U mL^−1^ penicillin and 100 μg mL^−1^ streptomycin at 37 °C and 5% CO_2_. CHO-K1 (3.0 × 10^4^ per well) were seeded into a 96-well plate and incubated overnight. The following day, the cells were treated with ten-fold serial dilutions of each construct (LDn1–11) in the presence of 20 nM PA for 30 minutes at 37 °C and 5% CO_2_. After treatment, the cells were washed three times with PBS and then treated with 100 μL leucine free medium containing 1 μCi mL^−1 3^H-Leucine (PerkinElmer) for 1 hour at 37 °C and 5% CO_2_. After treatment with ^3^H-Leu, the cells were washed three times with PBS and then suspended in 150 μL of scintillation fluid. ^3^H-Leu incorporation was measured using a scintillation counter to determine the inhibition of protein synthesis by LF_N_-DTA in the cytosol. Cells treated with PA only were used for normalization. Each experiment was done in triplicate. The data were plotted using Origin8 using Sigmoidal Boltzmann Fit using equation 
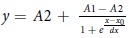
 and x_0_ represents the log(EC_50_) values.

### Translocation of LDn1–11 and cytosolic and total cell lysate extraction

CHO-K1 (2.0 × 10^5^ per well) were seeded in a 12-well plate and incubated overnight. The cells were treated with 100 nM LDn1–11, LDn1-bio to LDn6-bio, LDn9-bio, and LDn11-bio in the presence of 20 nM PA for 12 hours at 37 °C and 5% CO2. After treatment, the cells were lifted and the cell surface was digested with 0.25% trypsin-EDTA for 5 minutes. The cells were then washed twice with PBS. For cytosolic protein extraction, 0.5 × 10^5^ cells were resuspended in 50 μL of 50 μg mL^−1^ digitonin in 75 mM NaCl, 1 mM NaH_2_PO_4_, 8 mM Na_2_HPO_4_, 250 mM sucrose supplemented with Roche protease inhibitor cocktail for 10 min on ice, and centrifuged for 5 minutes at 13,000 rpm. For total protein extraction, cells were lysed in total cell lysis buffer (25 mM Tris, 150 mM NaCl, 1% v/v NP-40, pH 7.5) supplemented with Roche protease inhibitor cocktail on ice for 30 minutes and centrifuged for 10 minutes at 13,000 rpm. Both supernatants were collected for western blot.

### Western blot of extracted material

Extracted material was run on an SDS-PAGE gel (Life Technologies) for 35 minutes at 165 V and then transferred onto a nitrocellulose membrane soaked in 48 mM Tris, 39 mM glycine, 0.0375% SDS, 20% methanol and using a TE 70 Semi-Dry Transfer Unit (GE) for 1 hour at 17 V. The membrane was blocked at room temperature for 2 hours using LI-COR Odyssey blocking buffer (PBS). The membrane was immunostained overnight with anti-DTA, anti-LF, anti-Erk1/2, or anti-Rab5 in TBST at 4 °C. After incubation, the membrane was washed with TBST and incubated with the appropriate secondary antibody in TBST for 1 hour at room temperature then washed with TBST. The membrane was imaged by LI-COR Odyssey infrared imaging system.

## Additional Information

**How to cite this article**: Rabideau, A. E. *et al.* Translocation of Non-Canonical Polypeptides into Cells Using Protective Antigen. *Sci. Rep.* **5**, 11944; doi: 10.1038/srep11944 (2015).

## Supplementary Material

Supplementary Information

## Figures and Tables

**Figure 1:**
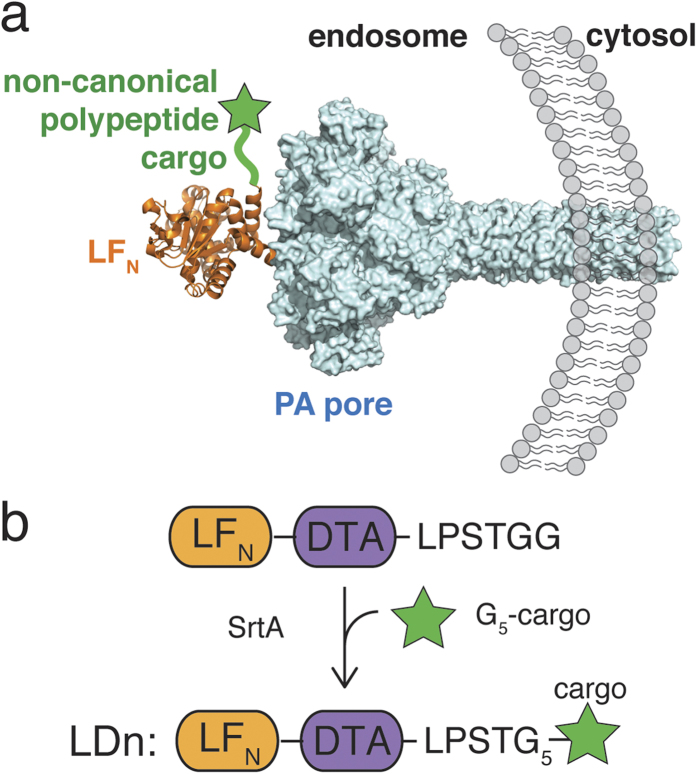
Delivery of non-canonical polypeptide cargo into the cytosol **a**) Non-canonical polypeptide cargo (green star) ligated to the C-terminus of LF_N_ (pdb: 1J7N) to translocate through PA pore (pdb: 3J9C) **b**) Sortase A-mediated ligation of LF_N_-DTA-LPSTGG and G_5_-cargo to form constructs are comprised of LF_N_-DTA and non-canonical polypeptide cargo (LDn).
