# Supplementary material for: Translocation of Non-Canonical Polypeptides into Cells Using Protective Antigen
Source: Sci Rep. 2015 Jul 16;5:11944. doi: 10.1038/srep11944 (PMC4503955; doi:10.1038/srep11944)

Translocation of Non-Canonical Polypeptides into Cells Using Protective Antigen

Amy E. Rabideau,[+] Xiaoli Liao,[+] Gizem Akçay, Bradley L. Pentelute*

Supplementary Information

Table of Contents

**Title**	**Page**
Materials	3
^{1}H Nuclear magnetic resonance (^{1}H NMR)	3
Synthesis of docetaxel-maleimide	3
Synthesis of doxorubicin-maleimide	3
Solid phase peptide synthesis (Boc)	4
Solid phase peptide synthesis (Fmoc)	4
Synthesis of docetaxel-maleimide	4
Synthesis of doxorubicin-maleimide	4
Protein expression and purification	5
Sortase-mediated ligation	5
Supplementary Table 1. Peptides used in this investigation	6
Supplementary Table 2. List of variants	7
Supplementary Table 3. EC50 values of 30-minute protein synthesis inhibition assay	9
Supplementary Scheme 1: Cyclization of L-linear peptide using native chemical ligation	10
Supplementary Scheme 2. Synthesis of doxorubicin-maleimide and docetaxel-maleimide	11
Supplementary Figure 1. Western blot of total extraction of LDn1-8	12
Supplementary Figure 2. Western blot of total extraction of LDn9-11	13
LC-MS Appendix	14

Materials

All chemicals were reagent grade and used as supplied except where noted. 2-(1H-Benzotriazol-1-yl)-1,1,3,3-tetramethyluronium hexafluorophosphate (HBTU), N-Fluorenyl-9-methoxycarbonyl (Fmoc) and di-tert-butyl-dicarbonate (Boc) protected amino acids were purchased from CreoSalus or Chem-Impex International. 4-Methylbenzhydrylamine (MBHA) resin was obtained from Anaspec, CA. N,N-dimethylformamide (DMF), dichloromethane (DCM), methanol (MeOH), diethyl ether, HPLC-grade acetonitrile (MeCN) and guanidine hydrochloride (guanidine·HCl) were from VWR. Trifluoroacetic acid (TFA) was purchased from NuGenTec and Sigma-Aldrich. 3-maleimidopropionic acid was purchased from Toronto Research Chemicals, Toronto, Ontario. Doxorubicin hydrochloride was purchased from AvaChem Scientific, docetaxel was purchased from A ChemTek Inc., and maleimido-monomethyl auristatin F (MMAF) was purchased from Concortis. All other reagents were purchased from Sigma-Aldrich and Life Technologies.

All moisture sensitive reactions were performed under argon atmosphere in oven-dried glassware and in anhydrous solvents. Reactions were monitored by thin-layer chromatography (TLC) carried on silica gel $60F_{254}$ plates (EMD Chemical Inc.) and were visualized under an UV lamp or by charring with phosphomolybdic acid or ninhydrin stain. Flash column chromatography was performed on 60Å silica gel (230-400 mesh) purchased from Whatman Inc.

The following primary and secondary antibodies were used goat anti-LF (bD-17, Santa Cruz Biotechnology), rabbit anti-DTA (ab8308 abcam), anti-Erk1/2 (137F5 cell signaling), anti-Rab5 (C8B1 cell signaling), goat anti-mouse IRdye 680RD (LI-COR Biosciences), goat anti-mouse IRdye 800CW (LI-COR Biosciences), goat anti-rabbit IRdye 800CW (LI-COR Biosciences), donkey anti-goat IRdye 680LT (LI-COR Biosciences), and streptavidin IRdye 680LT (LI-COR Biosciences).

^{1}H Nuclear magnetic resonance (^{1}H NMR): ^{1}H NMR spectra were obtained with a Bruker Advance III 400 MHz instrument and referenced to tetramethylsilane (TMS) at 0.00 ppm. Chemical shifts are recorded as parts per million (ppm) in δ scale and coupling constants J, are in hertz (Hz). Multiplicities are indicated as "s" (singlet), "bs" (broad singlet), "d" (doublet), "t" (triplet), "dd" (doublet of doublets), "ddd" (doublet of doublet of doublets), or "m" (multiplet).

Synthesis of docetaxel-maleimide

The identity of the product was confirmed by high resolution LCMS and ^{1}H-NMR. Calculated mass: 958.4 Da; observed $[H]^+$: 959.4 m/z.^{1}HMR (500 MHz, $CDCl_3$): δ 8.16 (d, J=7.55 Hz, C25, C29-H, 2H), 8.02 (s, 2H), 7.60 (t, J=7.30, 1H), 7.50 (t, J=7.6, 2H), 7.48-7.30 (m, 8H+$CDCl_3$), 6.70 (s, maleimide, CH, 2H) 6.58 (d, J=9.15 Hz, 2H) 6.15 (m, 2H), 5.75 (d, J= 9.80 Hz, 1H), 5.70 (d, J=6.95 Hz, C2-CH, 1H), 5.50 (bs, C3'-CH, 1H), 5.32 (bs, 1H), 5.26 (s, C10-CH, 1H), 4.88 (dd, J=8.20 Hz, C5-CH, 1H) 4.32 (d, J= 8.55 Hz, C20-CHb, 1H), 4.27 (m, C7-CH, 1H), 4.2 (d, J=4.75 Hz, C20-CH_a 1H), 3.95 (d, J=6.30 Hz, 1H), 3.85 (m, 1H), 3.70 (m, 1H), 3.50 (m, 7H), 2.55 (m, 2H), 2.48 (s, C22-CH_3, 3H), 1.98 (s, 2H), 1.90 (d, J=11.95 C14-$CH_{2,}$ 2H) 1.80 (s, C18-$CH_{3,}$ 3H), 1.72 (s, C19-$CH_{3,}$ 3H), 1.35 (9Hs of -*t*Bu), 1.22 (s, C16-CH_3, 3H), 1.10 (s, C17-CH_3, 3H)

Synthesis of doxorubicin-maleimide

The identity of the product was confirmed by MALDI and ^{1}H-NMR. Calculated mass: 694.2; Observed $[H+Na]^+$ 717.0 m/z and $[H+K]^+$ 732.9 m/z. ^{1}HMR (500 MHz, CH_3OD): δ 8.25 (d,

J=7.65 Hz, 1H), 8.00 (s, 1H), 7.85 (t, J=8.05 Hz, 1H), 7.61 (d, J=8.65 Hz, 1H), 6.78 (s, maleimide, CH, 2H), 5.42 (d, J=3.65 Hz, 1H), 5.21 (bs, 1H), 4.73 (d, J=3.5 Hz, 2H), 4.56 (s, CO-CH_2-OH, 2H), 4.25 (dd, J=6.75, 10.15 Hz, 2H), 4.08 (m, 2H), 4.05 (s, 3H, OCH_3), 3.72 (t, J= 6.95 Hz, 2H)) 3.60 (s, 1H), 3.44 (s, 2H), 3.20 (s, 2H), 3.12 (d, 2H), 2.90 (s, 2H), 2.45 (t, J=6.90 Hz, 2H) 2.40 (d, J=14.80 Hz, 2H) 2.20 (dd, J=4.80, 9.57 Hz, 2H), 1.90 (ddd, 1H), 1.68 (dd, J=4.35 and 12.95 Hz, 2H), 1.30 (s, 4H), 1.28 (d, J=6.60 Hz, 3H, CH_3)

Solid phase peptide synthesis (Boc)

Select peptides were synthesized using in situ neutralization boc chemistry. Peptides were synthesized on 0.2 mmol scale on MBHA resin and the following side chain protection was used for L- and D-amino acids: Arg(Tos), Asn(Xan), Asp(OcHex), Lys(2-ClZ), Lys(Alloc), and Ser(Bzl). For the cyclic peptides, peptide thioesters were prepared using the S-trityl mercaptopropionic acid (MPA) strategy. After peptide synthesis, the peptides were cleaved from the resin and side chains were deprotected using 10% (v/v) *p*-thiocresol and 10% (v/v) *p*-cresol in anhydrous HF for 1 h at 0 °C. Peptides were then triturated with cold diethyl ether, dissolved in 50:50 A:B (A: water + 0.1% TFA and B: acetonitrile + 0.1% TFA), and then lyophilized.

Solid phase peptide synthesis (Fmoc)

Select peptides were synthesized using fast flow Fmoc synthesis on a 0.1 mmol scale on aminomethyl resin with the Rink amide linker. Side-chain protection for the amino acids included: Arg(Pbf), Asn(Trt), Glu(OtBu), Lys(Boc), Lys(Alloc), Ser(tBu), Thr(tBu), Trp(Boc), and Tyr(tBu). After synthesis, peptides were cleaved from the resin with 94% TFA containing 2.5% EDT, 2.5% H_2O, and 1% TIPS (v/v) for 7 min at 60 °C. After cleavage, TFA was dried under N_2(g), triturated with cold diethyl ether, dissolved in 50:50 A:B, and then lyophilized.

The allyloxycarbonyl (Alloc) protecting group was removed using 4.85 mmol phenylsilane and 39.5 μmol tetrakis(triphenylphosphine)palladium(0) in DCM for 20 min at RT.[3] The resin was washed with DCM then DMF.

Synthesis of docetaxel-maleimide

Docetaxel (100 mg, 0.124 mmol) and maleimidopropionic acid (25 mg, 0.149 mmol) were taken in anhydrous DCM (1.5 mL), followed by addition of Mukaiyama's reagent; 2-chloro-1-methylpyridinium iodide (57 mg, 0.225 mmol) and excess triethylamine (0.2 mL) at 0°C. The reaction mixture was slowly warmed up to room temperature and stirred 16 hours, at which TLC analysis (5% v/v methanol in dichloromethane) indicated consumption of starting materials and formation of a major product. The reaction was quenched by addition of ethanol and additional stirring for 10 min, followed by concentration to dryness. The crude material was subjected to silica flash chromatography to give the thiol reactive docetaxel derivative, docetaxel-maleimide, 62 mg (52.2 % yield). The identity of the product was confirmed by high resolution LCMS and ^{1}H-NMR (supplementary information).

Synthesis of doxorubicin-maleimide

Doxorubicin (50 mg, 0.086 mmol) and N-succinimidyl ester of maleimidopropionic acid (45.78 mg, 0.172 mmol) were taken in DMF (1.6 mL) and reacted for 1 hour in the presence of N,N-diisopropylethylamine DIEA (50 μL), at which TLC analysis (20% v/v methanol in dichloromethane) indicated completion of the reaction and formation of a major product. The reaction mixture was quenched by diluting with DCM, followed by repetitive aqueous

extractions to remove DMF and unreacted doxorubicin. The combined organic phase was dried over magnesium sulfate ($MgSO_4$), inorganic salts were filtered off and concentrated in vacuo to dryness. The crude material was purified by silica flash chromatography (20% v/v methanol in dichloromethane) to give the thiol reactive doxorubicin derivative, doxorubicin-maleimide, 47.7 mg (80% yield). The identity of the product was confirmed by high resolution LCMS and ^{1}H-NMR (supplementary information).

Protein expression and purification

His_6-SUMO-LF_N-DTA(C186S)-LPSTGG-His_5, His_6-SUMO-LF_N-DTA(C186S), SrtA*-His_6, wild-type protective antigen (PA), and PA[F427H] were expressed in *E. coli* BL21 (DE3) cells at New England Regional Center of Excellence/Biodefense and Emerging Infectious Diseases (NERCE). Each His_6-tagged protein was purified using Ni-NTA columns. Each cell pellet (approximately 40 g) was resuspended in 100 ml of 50 mM Tris-HCl, 150 mM NaCl, pH 7.5 buffer containing 200 mg lysozyme, 4 mg Roche DNAase I, and 2 tablets of Roche protease inhibitor cocktail. The suspension was sonicated on ice three times for 20 seconds. After sonication, the suspension was centrifuged at 17,000 rpm for 40 minutes. The lysate was loaded onto three 5 ml GE HisTrap FF crude Ni-NTA column pre-equilibrated with binding buffer (20 mM Tris-HCl pH 8.5, 150 mM NaCl, at pH 8.5). After loading the lysate, the columns were washed with 100 mL binding buffer then 100 mL 40 mM imidazole in 20 mM Tris-HCl pH 8.5, 500 mM NaCl. The protein was eluted using 500 mM imidazole in 20 mM Tris-HCl pH 8.5, 500 mM NaCl. The eluted protein was buffer exchanged to remove the imidazole using a HiPrep 26/10 Desalting column (GE Healthcare, UK). Wild-type PA and PA[F427H] were overexpressed in the periplasm of *E. coli* BL21 (DE3) cells and purified by anion exchange chromatography.

Sortase-mediated ligation

Sortase A was used to ligate peptides containing the N-terminal oligoglycine motif to LF_N-DTA-LPSTGG (LDn). *Staphylococcus aureus* $^{59\text{-}206}$SrtA (P94S/D160N/K196T; SrtA*) evolved by Chen, et al. was used for our sortase-mediated ligations. In order to obtain a native N-terminus, the small ubiquitin-like modified (SUMO) was cleaved off LFN-DTA-LPSTGG. SUMO cleavage was achieved using 1 µg SUMO protease per mg of protein substrate at RT for 1 hour followed by gel or LCMS analysis to confirm complete cleavage. We perform the sortase-mediated ligations in the presence of Ni-NTA beads in order to bind all His_6-tagged reagents and release the His_6-free product in the supernatant. Ni-NTA beads were equilibrated with SrtA buffer (10 mM $CaCl_2$, 50 mM Tris-HCl, 150 mM NaCl, pH 7.5). In one pot, 50 µM LF_N-DTA-LPSTGG-His_5, 5 µM SrtA*, and 300 µM G_5-peptide were incubated with Ni-NTA beads in SrtA buffer for 30 min at RT while rotating. After incubation, the beads were spun down at 4 °C and the supernatant was collected. The beads were washed twice with SrtA buffer and twice with 10 mM imidazole in 20 mM Tris-HCl pH 7.5, 150 mM NaCl (to remove any non-specifically bound LF_N). The supernatant and all washes were combined, concentrated, and buffer exchanged three times into 20 mM Tris-HCl, 150 mM NaCl, pH 7.5 to remove the excess G_5-peptide. The purity of the ligated product (LDn) was analyzed by LCMS. Concentrations of the ligated products containing non-natural functionalities were determined using Bradford assay.

Supplementary Table 1. Peptides used in this investigation

Sequence	Observed (Da)	Calculated (Da; mono.)
**1** G_5-AKFRPDSNVRG-$CONH_2$	1529.8 ± 0.1	1529.8
**2** G_5-(β-Ala)KFRPDSNVRG-$CONH_2$	1529.8 ± 0.1	1529.8
**3** G_5-(N-Me-Ala)KFRPDSNVRG-$CONH_2$	1543.8 ± 0.1	1543.8
**4** G_5-(propargyl-Gly)KFRPDSNVRG-$CONH_2$	1553.8 ± 0.1	1553.8
**5** G_5-AK($PheF_3$)RPDSNVRG-$CONH_2$	1583.8 ± 0.1	1583.7
**6** G_5-AK(C*)FRPDSNVRG-$CONH_2$	1689.8 ± 0.1	1689.8
**7** G_5-AK(C*)FRPDSNVRG (cyclic)	1672.8 ± 0.1	1672.8
**8** (D)-G_5-AK(C*)FRPDSNVRG (cyclic)	1672.8 ± 0.1	1672.8
**9** G_5-LRRLRAC(doxorubicin)-$CONH_2$	1864.9 ± 0.1	1864.9
**10** G_5-LRRLRAC(docetaxel)-$CONH_2$	2129.0 ± 0.1	2129.0
**11** G_5-LRRLRAC(MMAF)-$CONH_2$	2095.2 ± 0.1	2095.2
**1-bio** G_5-AKFRPDSNVRGK(biotin)-$CONH_2$	1884.0 ± 0.1	1884.2
**2-bio** G_5-(β-Ala)KFRPDSNVRGK(biotin)-$CONH_2$	1884.0 ± 0.1	1884.2
**3-bio** G_5-(N-Me-Ala)KFRPDSNVRGK(biotin)-$CONH_2$	1898.0 ± 0.1	1898.0
**4-bio** G_5-(propargyl-Gly)KFRPDSNVRGK(biotin)-$CONH_2$	1908.0 ± 0.1	1908.0
**5-bio** G_5-AK($PheF_3$)RPDSNVRGK(biotin)-$CONH_2$	1938.0 ± 0.1	1938.0
**6-bio** G_5-AK(C*)FRPDSNVRGK(biotin)-$CONH_2$	1987.0 ± 0.1	1987.0
**9-bio** G_5-LRRLRAC(doxorubicin)K(biotin)-$CONH_2$	2219.1 ± 0.1	2219.0
**11-bio** G_5-LRRLRAC(MMAF)K(biotin)-$CONH_2$	2449.4 ± 0.1	2449.4

* : alkylation

Supplementary Table 2. List of variants

Variant	Sequence
LDn1	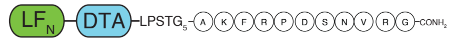
LDn2	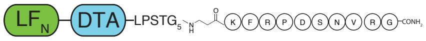
LDn3	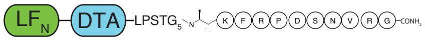
LDn4	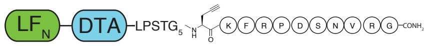
LDn5	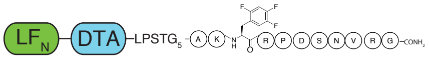
LDn6	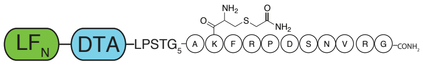
LDn7	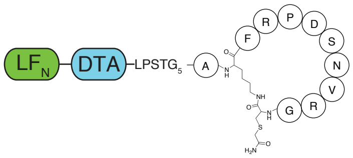
LDn8	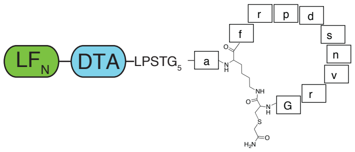
LDn9	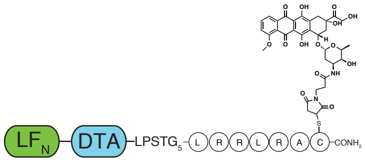
LDn10	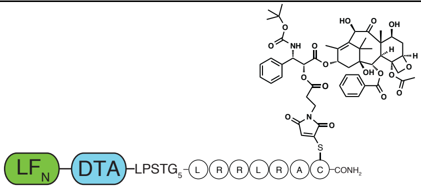
LDn11	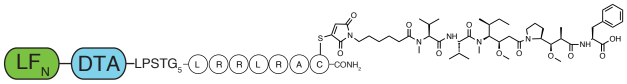
LDn1-bio	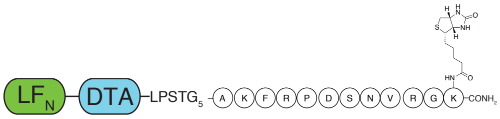
LDn2-bio	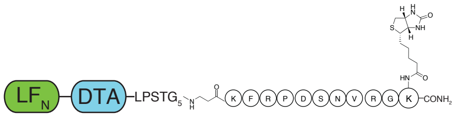
LDn3-bio	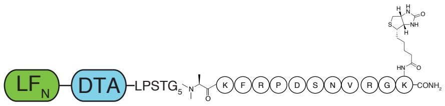

LDn4-bio	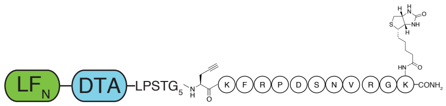
LDn5-bio	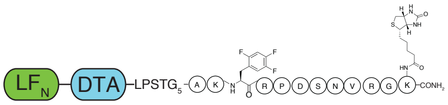
LDn6-bio	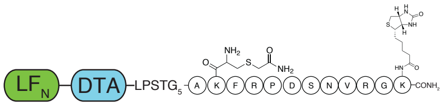
LDn9-bio	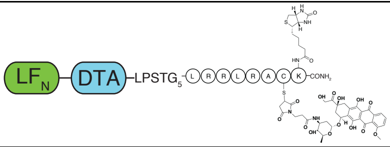
LDn11-bio	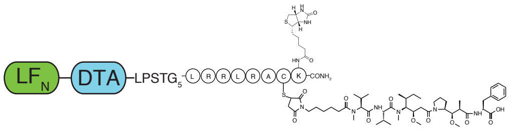

Supplementary Table 3. EC50 values of 30-minute protein synthesis inhibition assay. The errors represent fitting errors from Sigmoidal Boltzmann Fit. # represents data from a separate assay. n/a indicates that the data could not be properly fit with the Sigmoidal Boltmann Fit.

Protein	EC50 (pM)	Protein	EC50 (pM)
LF_N-DTA	21 ± 3	LF_N-DTA#	74 ± 12
LDn1	37 ± 5	LDn9#	128 ± 18
LDn2	31 ± 10	LDn10#	n/a
LDn3	31 ± 5	LDn11#	428 ± 90
LDn4	48 ± 15		
LDn5	24 ± 3		
LDn6	36 ± 5		
LDn7	n/a		
LDn8	n/a		

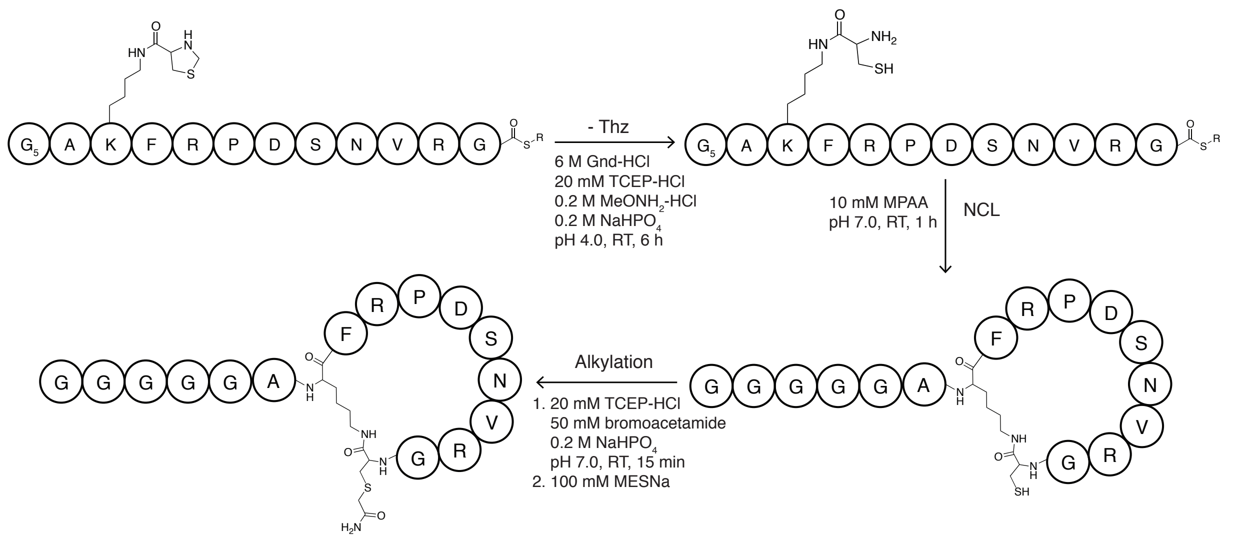

Supplementary Scheme 1: Cyclization of L-linear peptide using native chemical ligation

a)

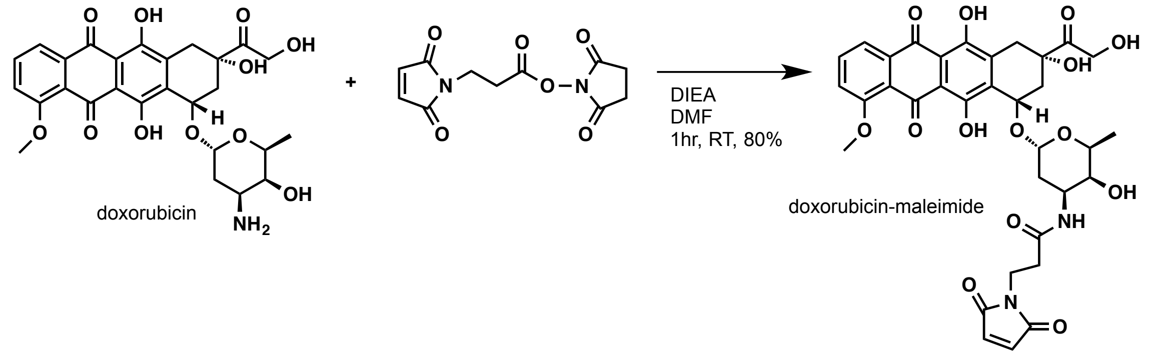

b)

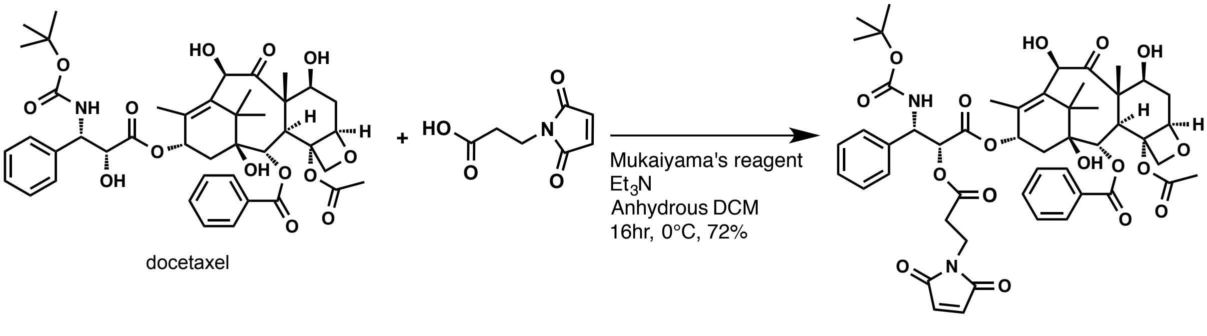

Supplementary Scheme 2. Synthesis of doxorubicin-maleimide (a) and docetaxel-maleimide (b).

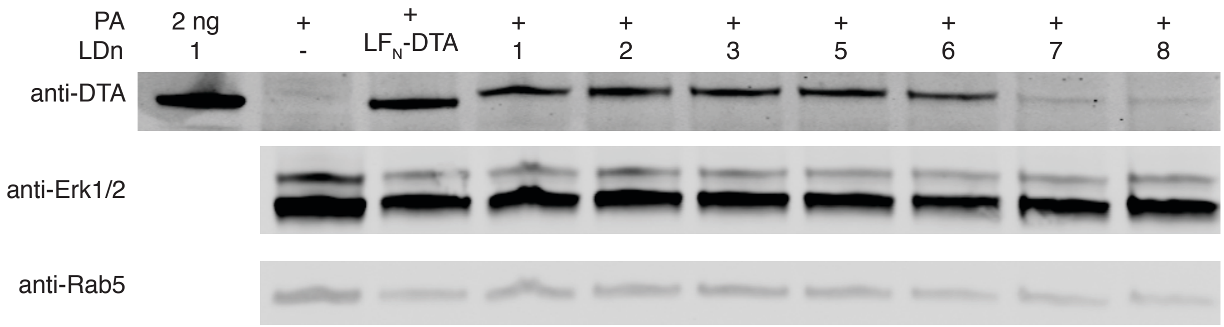

Supplementary Figure 1. Western blot of total extraction of LDn1-8. Cropped blots are used in western blot data.

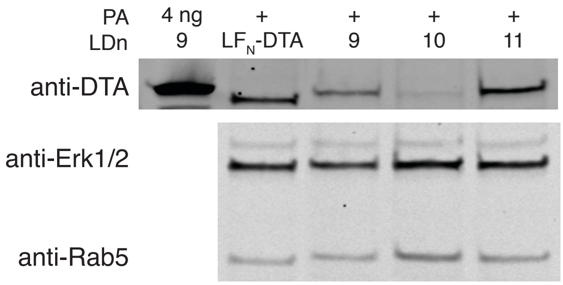

Supplementary Figure 2. Western blot of total extraction of LDn9-11. Cropped blots are used in western blot data.

LC-MS Appendix:

LDn1

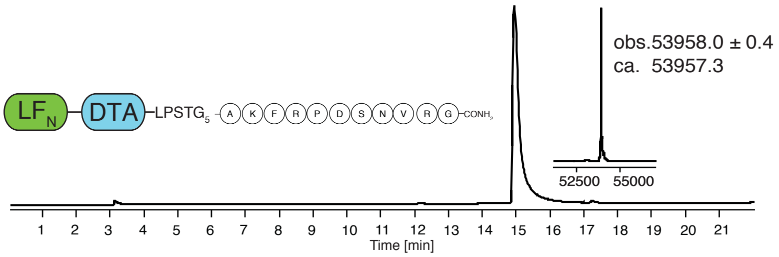

LDn2

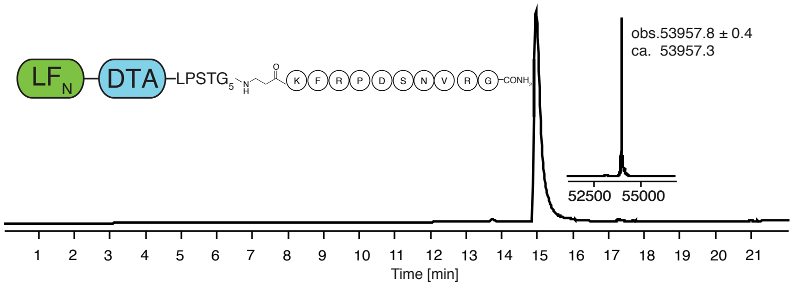

LDn3

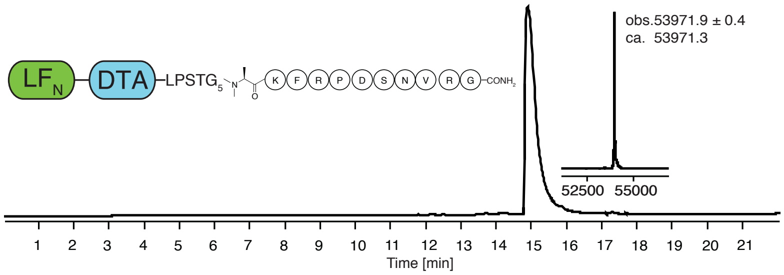

LDn4

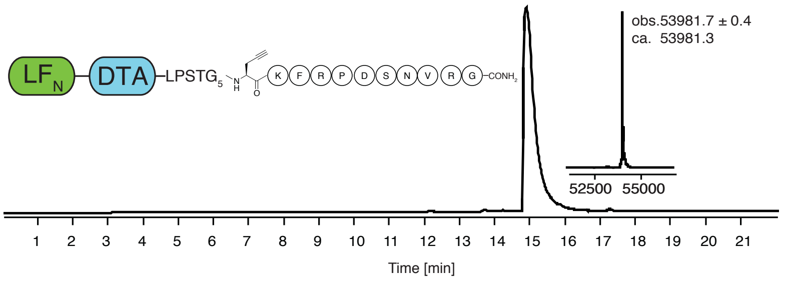

LDn5

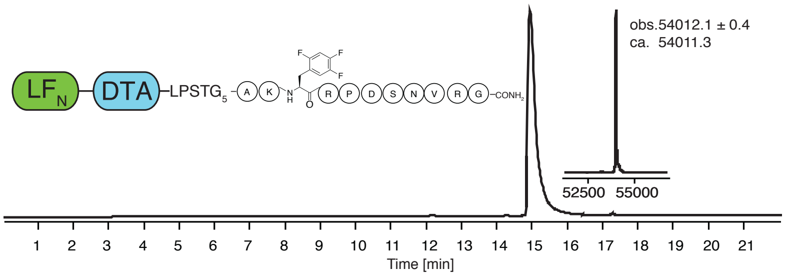

LDn6

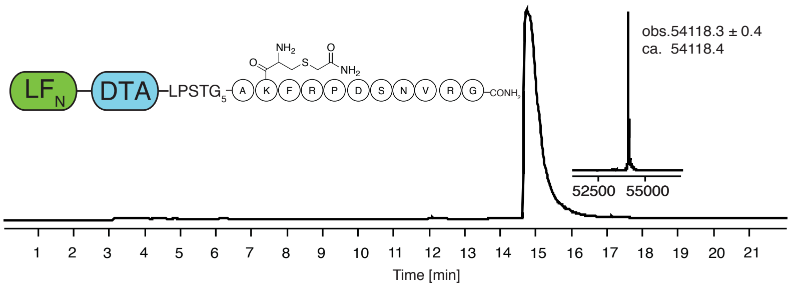

LDn7

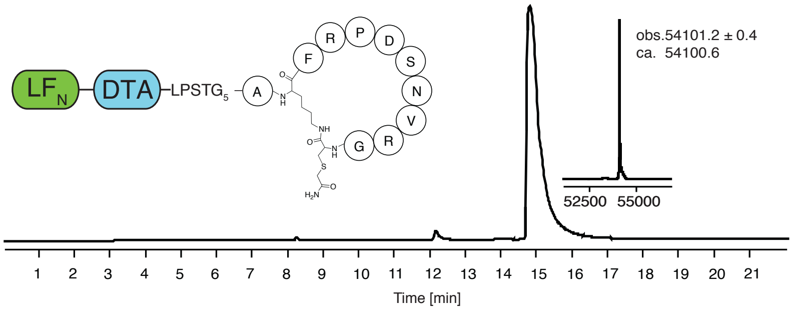

LDn8

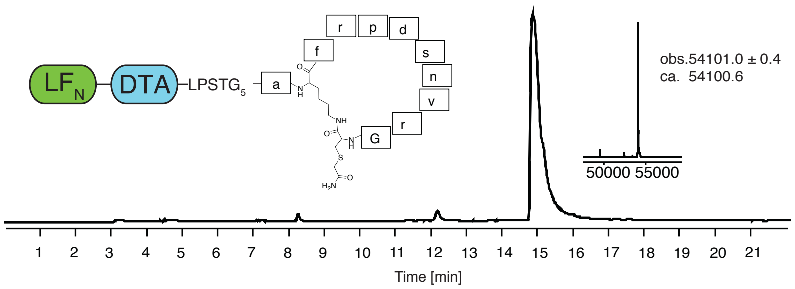

LDn9

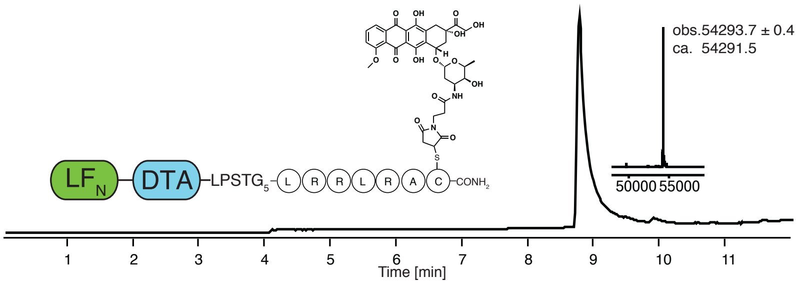

LDn10

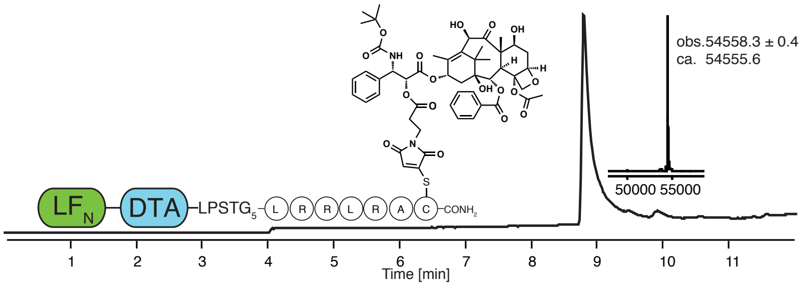

LDn11

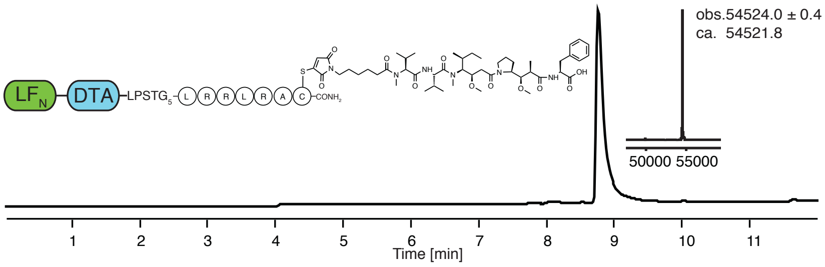

LDn1-bio

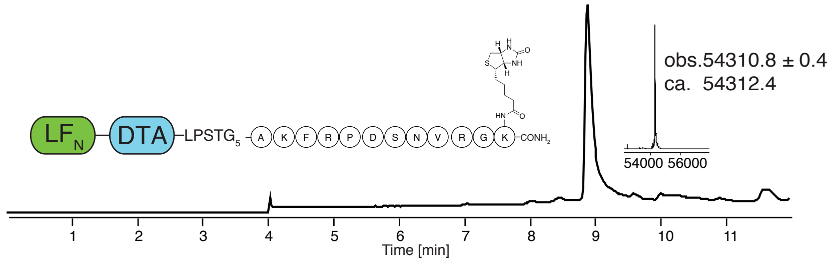

LDn2-bio

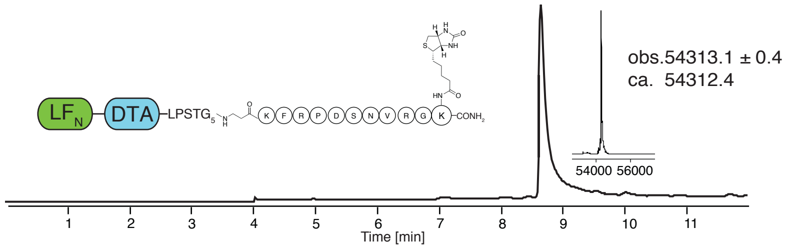

LDn3-bio

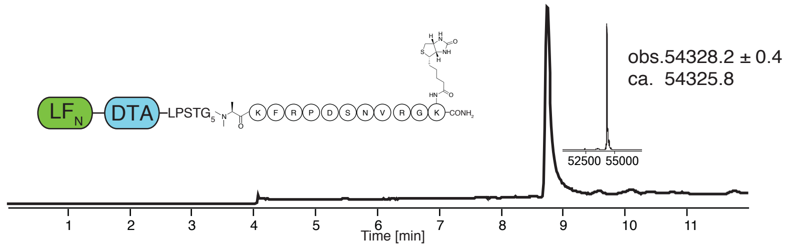

LDn4-bio

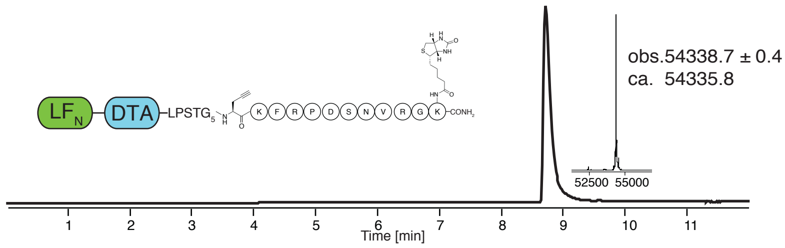

LDn5-bio

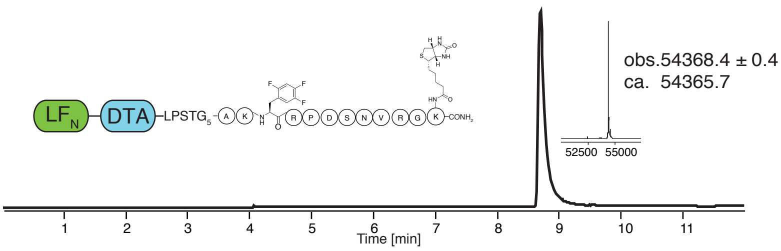

LDn6-bio

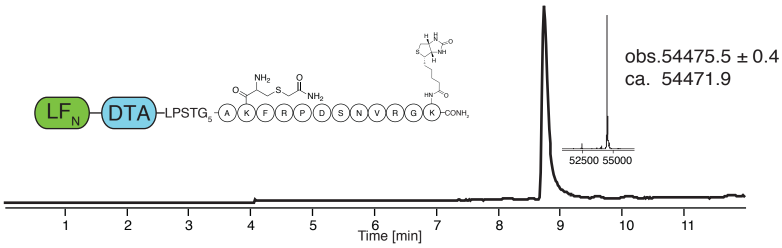

LDn9-bio

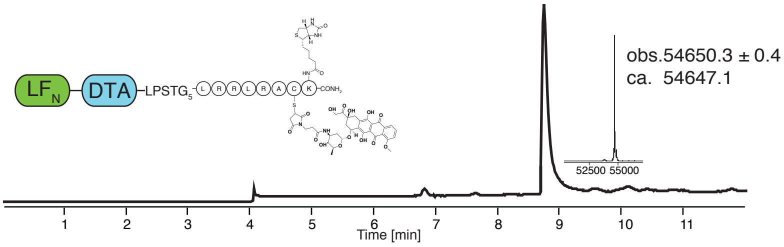

LDn11-bio

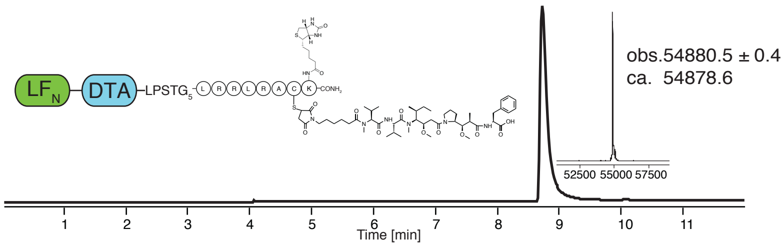

Supplement: Supplementary Information [file srep11944-s1.pdf]
